# Age-Dependent *in vitro* Maturation Efficacy of Human Oocytes – Is There an Optimal Age?

**DOI:** 10.3389/fcell.2021.667682

**Published:** 2021-06-18

**Authors:** Gilad Karavani, Peera Wasserzug-Pash, Talya Mordechai-Daniel, Dvora Bauman, Michael Klutstein, Tal Imbar

**Affiliations:** ^1^Fertility Preservation Service, Department of Obstetrics and Gynecology, Hadassah Ein Kerem Medical Center and Faculty of Medicine, The Hebrew University of Jerusalem, Jerusalem, Israel; ^2^Institute of Biomedical and Oral Research, Faculty of Dental Medicine, The Hebrew University of Jerusalem, Jerusalem, Israel

**Keywords:** *in vitro* maturation, ovarian tissue cryopreservation, fertility preservation, women age, oocyte

## Abstract

*In vitro* maturation of oocytes from antral follicles seen during tissue harvesting is a fertility preservation technique with potential advantages over ovarian tissue cryopreservation (OTC), as mature frozen and later thawed oocyte used for fertilization poses decreased risk of malignant cells re-seeding, as compared to ovarian tissue implantation. We previously demonstrated that *in vitro* maturation (IVM) performed following OTC in fertility preservation patients, even in pre-menarche girls, yields a fair amount of oocytes available for IVM and freezing for future use. We conducted a retrospective cohort study, evaluating IVM outcomes in chemotherapy naïve patients referred for fertility preservation by OTC that had oocyte collected from the medium with attempted IVM. A total of 133 chemotherapy naïve patients aged 1–35 years were included in the study. The primary outcome was IVM rate in the different age groups – pre-menarche (1–5 and ≥6 years), post-menarche (menarche-17 years), young adults (18–24 years) and adults (25–29 and 30–35 years). We demonstrate a gradual increase in mean IVM rate in the age groups from 1 to 25 years [4.6% (1–5 years), 23.8% (6 years to menarche), and 28.4% (menarche to 17 years)], with a peak of 38.3% in the 18–24 years group, followed by a decrease in the 25–29 years group (19.3%), down to a very low IVM rate (8.9%) in the 30–35 years group. A significant difference in IVM rates was noted between the age extremes – the very young (1–5 years) and the oldest (30–35 years) groups, as compared with the 18–24-year group (*p* < 0.001). Importantly, number of oocytes matured, percent of patients with matured oocytes, and overall maturation rate differed significantly (*p* < 0.001). Our finding of extremely low success rates in those very young (under 6 years) and older (≥30 years) patients suggests that oocytes retrieved during OTC prior to chemotherapy have an optimal window of age that shows higher success rates, suggesting that oocytes may have an inherent tendency toward better maturation in those age groups.

## Introduction

Cryopreservation of ovarian cortical tissue followed by auto-transplantation has emerged as a promising fertility preservation option (Feigin et al., 2007). Immature oocytes, usually in the germinal vesicle (GV) stage can be found during ovarian tissue handling (Revel et al., 2009; Gruhn et al., 2018) and the ability for maturation *in vitro* and cryopreservation as mature oocytes, provides an additional option for fertility preservation (Chian et al., 2013).

*In vitro* maturation (IVM) of oocytes from antral follicles seen during tissue harvesting is a fertility preservation technique with potential advantages over ovarian tissue cryopreservation (OTC). Although not well established, IVM is believed to be feasible even in the pediatric age group. A mature frozen and later thawed oocyte used for fertilization might serve as a safer option than re-implantation of ovarian cortex tissue, posing decreased risk of malignant cells re-seeding. In our previously published work, we demonstrated that IVM performed following OTC in fertility preservation patients, even in pre-menarche girls, yields a fair amount of oocytes available for IVM and freezing for future use. However, in the very young age group (under 6 years) there are substantially decreased maturation rates, making this procedure invaluable in this age group (Karavani et al., 2019).

The human age-specific fertility rate follows an inverted u shape curve: beginning to rise with puberty (usually marked by the occurrence of menarche), reaching a peak around the age of 30 years, followed by a decline, beginning around the age of 35 toward significant low maturity and fertility rate in the early 40s and later (Hawkes and Smith, 2010). A similar trend was observed for euploid oocyte rate across reproductive lifespan with a peak of euploid oocyte rate around the age of 25 (Gruhn et al., 2019). However, the rate of oocyte maturation capability as related to age has yet to be analyzed. Of important note is the fact that oocyte maturation capability is not necessarily linked to aneuploidy, and aneuploid oocytes can still mature.

As resources are limited and patients and their families are often required to fund the fertility preservation process, it is of need to optimize and personalize consultation regarding the success rate of IVM attempt in oocytes retrieved during OTC, especially considering the fact that an ovarian tissue with potential future use has already been preserved during the process.

Herein, we aimed to study the human age-specific oocytes maturation ability in naïve human oocytes collected from media of ovarian cortex pieces during fertility preservation procedures in order to define the age groups in which IVM should be attempted following OTC.

## Materials and Methods

### Study Population

Our study cohort consisted of female patients aged 1–35 years, referred to our fertility preservation clinic between 2003 and 2020. Patients for whom data regarding cancer diagnosis, chemotherapy, and OTC related data was available were included in the study. While those chemotherapy and radiotherapy exposed patients, and patients for which oocytes were not found in the medium following OTC (and therefore IVM was not performed) were excluded. Counseling regarding fertility preservation was done in a multidisciplinary consultation that included an oncologist, pediatric hematologist and reproductive specialist, as well as a coordinating nurse and social worker. Female patients under 18 years of age were consulted with parental presence. After obtaining informed consent, patients who decided to freeze ovarian tissue underwent partial/complete laparoscopic oophorectomy. In our country, OTC is reimbursed by the national health insurance and therefore does not require out of pocket coverage. The primary outcome were the overall and mean IVM rates in each age group.

### Data Collection

Data was collected from patients’ electronic medical records and from the IVF laboratory database. Parameters retrieved included: demographic and clinical data; cancer diagnosis; age at diagnosis; treatment with chemotherapy and its timing with regard to OTC; OTC procedure related information (partial or complete oophorectomy) and IVF laboratory data – including OTC (number of ovarian tissue ampules cryopreserved); oocytes retrieval and the IVM process and outcomes with emphasis given to the assessment of number of matured oocytes following IVM, overall and per patient IVM rate according to age groups and percent of patient with at least one matured oocyte following IVM.

### Ovarian Tissue Cryopreservation Procedure

The OTC procedure done in our center includes the removal of the entire (complete oophorectomy) or most of the ovary (partial oophorectomy) in a laparoscopic procedure. The tissue is then placed on ice in Leibovitz L-15 medium (GIBCO-BRL, Paisley, United Kingdom) and transferred immediately to the adjacent IVF laboratory for tissue examination and processing. In some cases, predominantly younger patients, insertion of a venous access device (port-a-cath) or bone-marrow aspiration is performed following the oophorectomy. In the case of normal post-operative course, discharge is customarily 24 h post procedure. Freezing of ovarian tissue has previously been described in the literature (Meirow et al., 1999) and performed in a similar fashion in our medical center. Briefly, ovarian cortex is carefully separated from the medulla in a specific media using sterile scissors. The tissue is then cut into 5-mm^3^ stripes, that are transferred to pre-cooled freezing medium containing 1.5 M dimethyl sulfoxide (DMSO) and 0.1 M sucrose. Each fragment is then placed in a 2 ml cryovial containing a cryoprotectant medium (sucrose, ethylene glycol, and serum substitute supplement) and processed using a slow freezing protocol in a programmable freezing machine (Kryo 360, Planer, United Kingdom). The frozen vials, each containing five cortex slices, are then stored in liquid nitrogen.

### Cumulus Oocyte Complexes Collection From the Media and Cortex

Following the cortex dissection and cryopreservation, the remaining medium is scanned for the presence of follicles and oocytes prior to disposal. Additionally, aspiration of antral follicles observed on the ovarian surface is performed under a dissecting microscope using a 1-mL syringe and a 19-gage needle prior to cryopreservation. Follicles found in the media and aspirated follicles are flushed with HEPES-buffered human tubal fluid (HTF) medium (Irvine Scientific, Santa Ana, CA, United States) containing 10% synthetic serum supplement (SSS; Irvine Scientific). All cumulus oocyte complexes collected are incubated in P1 medium (Irvine Scientific) supplemented with 10% SSS, at 37°C in 90% N_2_, 5% CO_2_, and 5% O_2_.

### *In vitro* Maturation

The IVM technique in our medical center has been previously reported (Karavani et al., 2019) and will be briefly described. IVM was performed on GV oocytes using Sage medium (Al-rad medical, Nes Ziona, Israel) supplemented with 0.075 IU/mL luteinizing hormone (LH) and 0.075 IU/mL follicle stimulating hormone (FSH) overnight. SAGE IVM culture media was used for the incubation of Cumulus-oocyte complexes, and 24-h later denudation of oocytes was performed. Additional 24 h incubation with fresh SAGE IVM culture media was applied for those oocytes which did not reach maturity. Finally, Matured MII oocytes were vitrified using Sage protocol.

### Study Age Groups

Patients included in the study (1–35 years) were divided into six groups, according to age at OTC. The pre-menarche patients were allocated to one of two groups – those under 6 years and those 6 years or older, as previous studies (Karavani et al., 2019; Fouks et al., 2020) found differences in IVM efficacy between these age groups. Post-menarche patients were divided into groups using 5-year intervals. Hence, the six age groups for this study were: (1) 1–5 years; (2) 6 years to menarche; (3) menarche to 17 years; (4) 18–24 years; (5) 25–29 years; (6) 30–35 years. The OTC and IVM outcomes were compared between each group. Additionally, statistical analysis was performed on the entire study cohort to evaluate possible parameters associated with the number of oocytes matured and the mean IVM rate per patient.

The Institutional ethical review board approval was received for this study (IRB 0288-16-HMO) by the Human Research Ethics Committees of the Hadassah Hebrew University Medical Center, Jerusalem, Israel.

### Statistical Analysis

Patient characteristics were described as proportions for categorical variables and the significance between groups was tested using the Fisher’s exact test. For quantitative variables [presented as mean ± standard deviation (SD)], the comparison between independent variables of the six study groups was performed using the Kruskall–Wallis test. Comparison between paired groups for significant difference in the IVM rate parameter was done using the Tukey’s Studentized Range (HSD) test. Association of the patients’ age and the *in vitro* maturation rate was tested using the Pearson correlation coefficient (“*r*”) for the 1–17 years and the 18–35 years age groups separately.

The ANCOVA analyses were applied to assess the effect of several parameters on the continuous dependent variables of mean IVM rate and number of oocytes matured using IVM. All tests applied were two-tailed, with a *p*-value of <0.05 considered statistically significant. SAS Version 9.4 (SAS Institute, Cary, NC, United States) software was used for statistical analysis.

## Results

### Study Population

A total of 202 patients were referred for fertility preservation by OTC in our fertility preservation unit, with planned attempted IVM process in cases of oocytes retrieved from the medium or the ovarian tissue. Following OTC, oocytes were retrieved and IVM was performed for 170 of 202 patients (87.0%), for which oocytes were available following retrieval from the medium. Thirty-seven patients received chemotherapy prior to OTC (21.8%) and were subsequently excluded, with a total of 133 patients (78.2%) being eligible and included in the final analysis. Age range was between 1 and 35 years, with a mean age of 17.15 ± 7.8 years [median 17 (IQR 10.5) years]. The most common indication for fertility preservation was diagnosis of hematologic conditions (34.6%), followed by sarcoma (30.8%), carcinoma and other solid tumors (24.1%), and non-oncological conditions (10.5%) (genetic, autoimmune, etc.). The mean age of menarche was 12.9 years. Oocytes were successfully matured in 68.4% (91/133) of patients, with an overall IVM rate for the entire study cohort being 25.0% (342/1370).

### Comparison of OTC Indication and Data According to Age Groups

The types of diagnoses leading to OTC in the patients included in the study are presented in Table 1. Solid tumors and sarcomas were the common cause for fertility preservation in the younger age group, prior to menarche (65%). This trend changes toward sarcomas and hematologic cancers from the age of menarche through 25 years of age (79.4%). Hematologic cancers are still common until the age of 30 (50%), but decline thereafter leaving solid tumors, mainly breast cancer, as the leading cause of fertility preservation in the over 30 years age group.

**TABLE 1:** Comparison of basic characteristics of the study population according to age groups (*n* = 133).

	1–5 years (*n* = 13)	6 years-menarche (*n* = 27)	Menarche-17 years (*n* = 35)	18–24 years (*n* = 33)	25–29 years (*n* = 14)	30–35 years (*n* = 11)	*p*-value
Age (years)	4.2 ± 1.4	10.3 ± 1.6	15.5 ± 1.4	20.4 ± 2.0	26.9 ± 1.2	32.5 ± 1.8	
Type of malignancy							<0.05^a^
Hematologic	0	7 (25.9%)	15 (42.9%)	15 (45.5%)	7 (50.0%)	2 (18.2%)	
Solid/carcinoma	7 (53.8%)	4 (14.8%)	6 (17.1%)	4 (12.1%)	4 (28.6%)	7 (63.6%)	
Sarcoma	3 (23.1%)	12 (44.4%)	13 (37.1%)	11(33.3%)	1 (7.1%)	1 (9.1%)	
Vascular/neurologic/other	3 (23.1%)	4 (14.8%)	1 (2.9%)	3 (9.1%)	2 (14.3%)	1 (9.1%)	
Partial oophorectomy	3 (23.1%)	15 (55.6%)	13 (37.1%)	8 (24.2%)	1 (7.1%)^b^	0^b^	0.002

*Data are given as mean ± SD, *n* (%) or *n*/*N* (%).*

*^*a*^Tukey’s Studentized Range test showed a significant difference only between the 1–5 years and the menarche-17 years groups.*

*^*b*^Tukey’s Studentized Range test showed a significant difference when compared to the 6 years-menarche group.*

Throughout all age groups, nearly seventy percent of the patients underwent total unilateral oophorectomy, with different rates of partial oophorectomy (*p* = 0.002), the lowest being in the age extremes [1–5 years (23.1%) and 25–35 years (4%)].

### OTC Results and IVM Outcomes in the Different Age Groups

**FIGURE 1:**
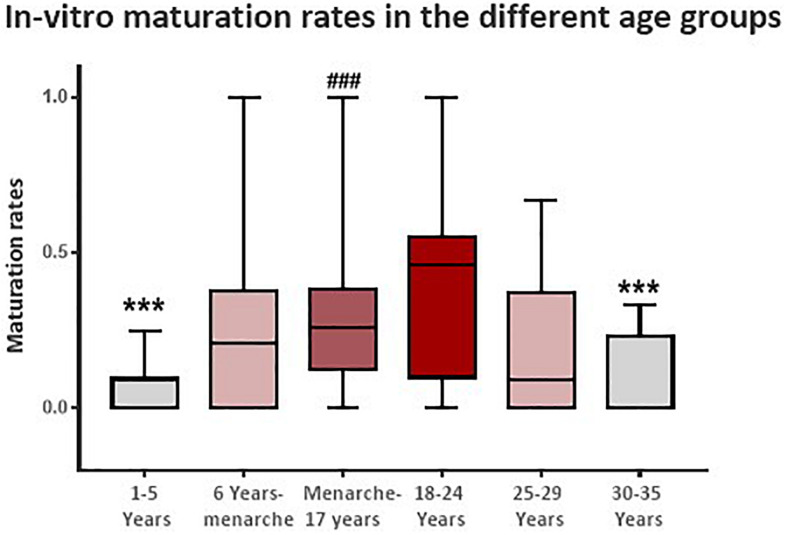
Patient *in vitro* maturation rate following ovarian tissue cryopreservation according to six age groups – (1) 1–5 years; (2) 6 years to menarche; (3) menarche to 17 years; (4) 18–24 years; (5) 25–29 years; and (6) 30–35 years. ***p < 0.001 when compared to the 18–24 years age group. ^###^*p* < 0.001 when compared to the 1–5 years age group.

Ovarian tissue cryopreservation procedure results in each age group are presented in Table 2. The number of ampules with ovarian cortex tissue preserved per patient differed significantly with age (*p* < 0.001), showing an increased number of ampules preserved at advanced patient age, with a significantly lower yield of cortical stripes in the very young (under the age of 6) age group. Interestingly, the number of oocytes found per patient during the procedure is similar throughout all the different age groups (*p* = 0.073). However, the similar oocytes harvest was not reflected in the total number of oocytes eventually matured and frozen. The oocytes from the youngest age group (1–5 years) demonstrated a low maturation rate, with the mean number of oocytes matured in patients under the age of 6 being only 0.4 with and an overall IVM rate of only 4.6%. The number of mature oocytes and total IVM rate increased with patient age, reaching its peak in the 18–24 age group (3.6 oocytes matured and 38.1% IVM rate). This peak was followed by an abrupt decrease in mean IVM rates in the 25–29 years group (19.3%), down to an extremely low mean IVM rate of 8.9% in the 30–35 years group (Table 2 and Figure 1). In a paired group analysis, corrected to multiple comparisons [Tukey’s Studentized Range (HSD) test], a significant difference in IVM rates was noted between the age extremes – the very young (1–5 years) and the oldest (30–35 years) groups as compared with the 18–24 years (*p* < 0.001). Number of oocytes matured, percent of patients with at least one matured oocyte and the overall maturation rate showed a similar pattern with a significant difference between the age groups (*p* < 0.001 for all three parameters) (Table 2).

**TABLE 2:** Comparison of ovarian tissue cryopreservation and *in vitro* maturation results according to age groups (*n* = 133).

	1–5 years (*n* = 13)	6 years-menarche (*n* = 27)	Menarche-17 years (*n* = 35)	18–24 years (*n* = 33)	25–29 years (*n* = 14)	30–35 years (*n* = 11)	*p*-value
No. of ampules preserved^a^	8.2 ± 1.9^b^	10.4 ± 2.6^c^	12.9 ± 2.6	14.2 ± 3.0	14.0 ± 2.8	12.3 ± 3.4	<0.001
No. of oocytes retrieved per patient	9.0 ± 7.0	11.2 ± 6.2	11.4 ± 8.5	10.5 ± 7.4	9.9 ± 11.7	6.0 ± 6.4	0.073
No. of oocytes matured per patient	0.4 ± 0.7^d,e^	2.4 ± 2.4	3.4 ± 3.3	3.6 ± 3.3	2.1 ± 3.5	0.5 ± 0.9^d^	<0.001
No. of patients with matured oocytes	4 (30.8%)^e^	20 (74.1%)	30 (85.7%)	26 (78.8%)	7 (50.0%)	4 (33.3%)	<0.001
Maturation rate per patient (%)	4.6 ± 0.1^d^	23.8 ± 22.2	28.4 ± 21.5	38.3 ± 26.8	19.3 ± 19.3	8.9 ± 12.3^d^	<0.001
Overall maturation rate	5/117 (4.3%)^f^	65/302 (21.5%)	118/398 (29.7%)	118/348 (32.9%)	30/139 (21.6%)	6/66 (9.1%)^g^	<0.001

*Data are given as mean ± SD, *n* (%) or *n*/*N* (%).*

*^*a*^Reflecting tissue volume. Each ampule contains 10 slivers of ovarian tissue for grafting.*

*^*b*^Tukey’s Studentized Range test showed a significant difference (*p* < 0.001) when compared to the menarche – 35 years age groups.*

*^*c*^Tukey’s Studentized Range test showed a significant difference (*p* < 0.001) when compared to the menarche – 29 years age groups.*

*^*d*^Tukey’s Studentized Range test showed a significant difference (*p* < 0.001) when compared to the 18–24 years group.*

*^*e*^Tukey’s Studentized Range test showed a significant difference (*p* < 0.001) when compared to the menarche-17 years group.*

*^*f*^Tukey’s Studentized Range test showed a significant difference (*p* < 0.001) when compared to the 6–29 years age groups.*

*^*g*^Tukey’s Studentized Range test showed a significant difference (*p* < 0.001) when compared to the menarche-17 and 18–24 years age groups.*

**FIGURE 2:**
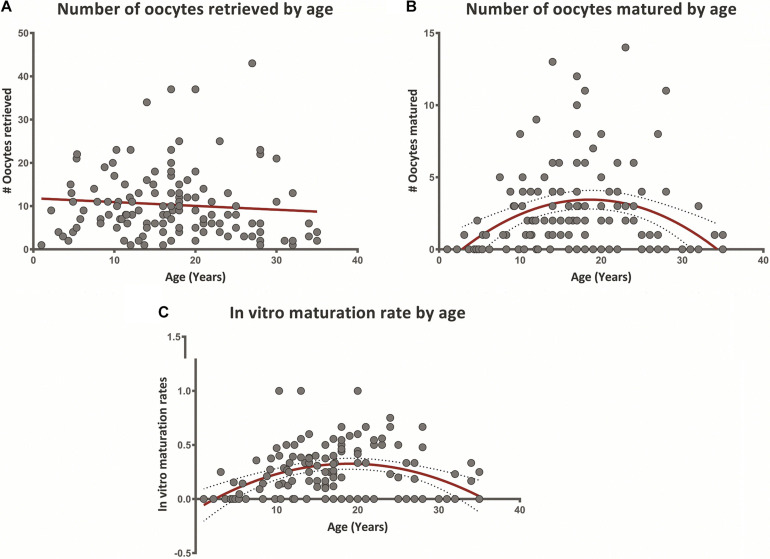
**(A–C)** Oocyte retrieval following ovarian tissue cryopreservation and *in vitro* maturation outcomes according to patient age (1–35 years). **(A)** Number of oocytes retrieved following ovarian tissue cryopreservation according to patient age. Linear regression trend is observed in red. **(B)** Number of oocytes matured by *in vitro* maturation according to patient age. Quadric fit line presented in red, with confidence interval of 95% (dotted lines). **(C)** Average rate of mature oocytes/total oocytes retrieved (*in vitro* maturation rate) per patient according to age. Quadric fit line presented in red, with confidence interval of 95% (dotted lines).

Figures 2A–C presents a cross-sectional association of patient age with the number of oocytes retrieved, matured, and *in vitro* maturation rates. The number of oocytes retrieved showed no significant trend with age (Figure 2A), a result consistent with the data presented in Table 2. Quadric fit was calculated for the number of mature oocytes retrieved from each patient, presenting negative orientation, with peak at the age of 18.5 (Figure 2B). The same process was applied to maturation rates, with the percent of mature oocytes retrieved from total oocytes pool per patient. The quadric equation presented negative orientation for the maturation rates as well, with a peak detected at the age of 18 (Figure 2C). To further asses the presence of the increase and subsequent decline of maturation potential, linear correlation between age and number of mature oocytes retrieved and IVM rate was performed for patients aged 1–17 years and aged 18–35 years separately (according to the trend demonstrated throughout the age groups). This analysis showed a significant positive linear correlation between patients aged 1–17 years for both parameters – (*r* = 0.36 and *r* = 0.31, *p* = 0.017 and *p* = 0.006; respectively), while demonstrating an inverse, negative correlation between age and number of mature oocytes in older patients – the 18–35 year group (*r* = −0.33 and *r* = −0.38, *p* = 0.012 and, *p* = 0.004; respectively).

Performing ANCOVA analyses, we did not find that type of malignancy, type of oophorectomy (partial/complete), age at diagnosis (as a continuous parameter) or number of oocytes retrieved to be significantly associated with the IVM rate.

## Discussion

This study aimed to evaluate the efficacy of *in vitro* maturation following OTC in a large cohort of patients of different ages from infancy to adulthood. The main outcome was the average IVM rate and the overall IVM rate according to age at OTC, using 5-year intervals and consideration of menarche status.

Our results show that while the number of oocytes retrieved during OTC without any prior external gonadotropin priming is similar in the different age groups, the ability to mature these oocytes *in vitro*, is related to patient age. We found that the optimal age for maximal IVM efficacy – represented by the highest IVM rates – is between menarche to 25 years (29–38% maturation rate), while young pre-menarche girls (under 6 years) and women 30 years or older achieve extremely low (<10%) IVM rates This trend was also seen in other parameters, such as the number of matured oocytes, percent of patients with matured oocytes and the overall maturation rate within each age group.

Interestingly, our findings suggest that the potential of immature oocytes to mature *in vitro* continuously improves during childhood, even prior to menarche. Later in life, the loss of *in vitro* maturation potential begins as early the as the mid-twenties with a sharp decline during the first half of the 4th decade of life. These findings imply that ovarian aging as expressed by the ability to mature unstimulated oocytes starts earlier than we know from our clinical results in the IVF settings, whereas pregnancies and live birth rates tend to dramatically drop along the fifth decade of life (Broekmans et al., 2007). These maturation defects can stem from many different biological pathways and processes, which may include: a change in maturation-related ovarian processes that are altered with age (Mara et al., 2020); changes in the composition and behavior of the ovarian tissue with age (Amargant et al., 2020); epigenetic changes that occur in oocytes with age (Wasserzug-Pash and Klutstein, 2019; Wasserzug-Pash et al., 2020) and enhanced ROS (Di Emidio et al., 2014; Babayev et al., 2016) and DNA damage (Marangos et al., 2015; Wasserzug-Pash and Klutstein,2019) in older oocytes. The results shown here emphasize that aged oocytes begin to deteriorate even before the onset of significant aneuploidy, as demonstrated in mouse oocytes (Merriman et al., 2012).

These results also show that young oocytes have a reduced maturation capacity before the onset of menarche. The improvement of oocyte maturation and quality in the younger age groups may also stem from several possible mechanisms. Although the specific reasons for this phenomenon are unknown, such mechanisms may include epigenetic changes occurring in oocytes with age (Wasserzug-Pash and Klutstein, 2019), and differential gene expression with age (Reyes et al., 2017). These finding may also imply that handling and culturing of oocytes from infants and older women should be managed through a different IVM technique and not the standard, widely used laboratory IVM protocol, with consideration of the different physiology and mechanisms involved in oocyte maturation in these age groups. It should be mentioned that the properties and behavior of oocytes in very young ages are an under-studied subject that merits more attention.

Previous studies evaluating the effect of patient age on oocyte maturation and fertilization ability had demonstrated that natural fertility in humans follows an inverse U-curve, where young females (≥13 to early 20s) and women of advancing maternal age (mid-30s and above) show reduced rates of pregnancy and live births (Hawkes and Smith, 2010; Fouks et al., 2020). This inverted U phenomena was recently attributed to chromosome segregation in human oocytes causing increased rates of oocyte aneuploidy in both ends of the fertility life span. The authors suggested that chromosome-based mechanisms in oocytes determines the curve of natural fertility in humans, and that the reasons for aneuploidy differ between young and old oocytes (7). While our findings agree with the presence of an inverted U-curve in human fertility, in our fertility preservation patients the peak of the curve is shifted to the left, toward the younger age where naïve oocytes have the ability for IVM. In our study, we found a peak of maturation is achieved around the 18–24-year age group, as compared to a peak age of 28 for euploid oocytes as demonstrated by Gruhn et al. (2019). These clinical findings may imply that other factors aside from chromosome number influences natural fertility span as suggested above.

This study has several limitations, the main being its retrospective design. Moreover, data regarding ovarian reserve evaluation was not available for most of the patients, due to our pre-op OTC procedures protocol. Up to date, none of our patients have used their frozen *in vitro* matured oocytes, and as such, further implications of age on *in vitro* matured oocytes quality and implantation potential have yet to be evaluated. As far as we know, this study is the largest cohort that evaluated patients that underwent IVM in the context of fertility preservation.

The strengths of this study include the cohort’s wide age range (1–35 years), enabling us to compare IVM efficacy in a single center according to different age groups. Patients were not exposed to any external gonadotropins prior to searching and handling the oocytes, thereby enabling us to study the unperturbed IVM rate of oocytes for each patient naturally. In addition, all oocytes were evaluated and treated in the same center using a single IVM protocol, performed by the same trained staff of embryologists, thereby minimizing the laboratory technique effect on the general results.

## Conclusion

To conclude, the ability to mature *in vitro* oocytes retrieved during OTC is associated with patient age. The optimal IVM efficacy is prior to menarche until 25 years of age. Young pre-menarche girls and women over age 30 demonstrated extremely low (<10%) IVM success rates. IVM of oocyte retrieved from patients in these age groups should be considered carefully, with regard to its potential efficacy and possible alternative laboratory approach should be investigated. Our results show that oocyte maturation has an optimal age window in which the success rate is much higher-demonstrating higher competence of oocytes in this window.

## Data Availability Statement

The original contributions presented in the study are included in the article/supplementary material, further inquiries can be directed to the corresponding author/s.

## Ethics Statement

The studies involving human participants were reviewed and approved by The Institutional Ethical Review Board approval was received for this study (IRB 0288-16-HMO) by the Human Research Ethics Committees of the Hadassah Hebrew University Medical Center, Jerusalem, Israel. Written informed consent to participate in this study was provided by the participants’ legal guardian/next of kin.

## Author Contributions

GK and PW-P contributed to the conception and design of the study, analysis and interpretation of the data, and drafting of the manuscript. TM-D contributed to the acquisition, analysis, and interpretation of data and drafting and revision of the manuscript. DB contributed to the interpretation of the data and drafting and revision of the manuscript. MK and TI contributed to the conception and design of the study, acquisition, analysis, and interpretation of the data, and drafting and revision of the manuscript. All authors have approved the final version of the study.

## Conflict of Interest

The authors declare that the research was conducted in the absence of any commercial or financial relationships that could be construed as a potential conflict of interest.
